# Dissimilar Joining Technologies for Aluminum and Steel: Interface Regulation, Defect Suppression, and External Field Assistance

**DOI:** 10.3390/ma19214499

**Published:** 2026-10-26

**Authors:** Zelong Wang, Feng Guo, Wenxiang Du, Fadhlan Mohd Yusof, Mahadzir Ishak

**Affiliations:** 1 Faculty of Mechanical and Automotive Engineering Technology, Universiti Malaysia Pahang Al-Sultan Abdullah, Pekan 26600, Pahang, Malaysia; wangzelong1003@163.com (Z.W.);; 2 School of Mechanical Engineering, Shandong Huayu University of Technology, Dezhou 253000, China; 3 Engineering Training Center, Shandong Huayu University of Technology, Dezhou 253000, China

**Keywords:** aluminum, steel, dissimilar material joining, thermal joining, mechanical joining, composite joining

## Abstract

In the automotive manufacturing industry, lightweighting has emerged as a key strategy for improving energy efficiency and reducing greenhouse gas emissions. Automobile manufacturers have successively adopted the strategy of replacing steel with aluminum (Al) and using higher-strength steel (HSS) to achieve vehicle weight reduction. However, Al and steel have significant differences in thermophysical properties, which greatly increases the difficulty of joining them. Numerous studies have effectively controlled the thickness of the intermetallic compounds (IMCs) layer at the weld interface by optimizing process parameters, adding filler materials and interlayers, and applying external energy fields. Therefore, this review categorizes Al/steel joining technologies into thermal, mechanical, and composite joining. It systematically discusses and summarizes the latest research findings, current applications, and development trends for each joining method.

## 1. Introduction

In recent years, energy conservation and emission reduction have become necessary responsibilities for all industries [1]. Within the automotive manufacturing industry, lightweighting has attracted growing attention [2]. Studies demonstrate that a 10% reduction in vehicle mass can result in a corresponding decrease in fuel consumption of approximately 7% [3,4]. Accordingly, automotive manufacturers are increasingly adopting optimized structural designs and advanced lightweight materials to reduce vehicle mass without compromising safety.

High-specific-strength materials such as aluminum (Al) alloys, magnesium (Mg) alloys, and higher-strength steel (HSS) have gained widespread adoption in various fields due to their superior properties [5,6,7]. In particular, Al alloys have become one of the most important lightweight materials in the automotive industry because of their low density and excellent corrosion resistance. To reduce vehicle weight and improve fuel efficiency, the “replace steel with Al” strategy has been increasingly adopted [8]. This approach expands the scope of Al alloys to reduce the overall vehicle weight, while HSS is still retained in critical structural components to ensure the required strength and stiffness.

The rise in multi-material hybridization offers new approaches to vehicle lightweighting. Their respective advantages can be fully leveraged by rationally combining materials with different properties [9]. This achieves comprehensive performance enhancements in strength, toughness, and weldability. For instance, the body-in-white of the new energy vehicles features an Al/steel structure, with Al on the bottom and steel on top. It significantly reduces the vehicle’s weight as the proportion of aluminum alloy in the material composition continues to increase. The press hardened steel (PHS) is used to enhance strength and stiffness for structural support parts such as the A-pillars and B-pillars [10]. Conversely, the die-cast Al alloys are employed for crumple zones such as the front and rear bumpers to improve toughness and energy absorption capacity.

**Figure 1:**
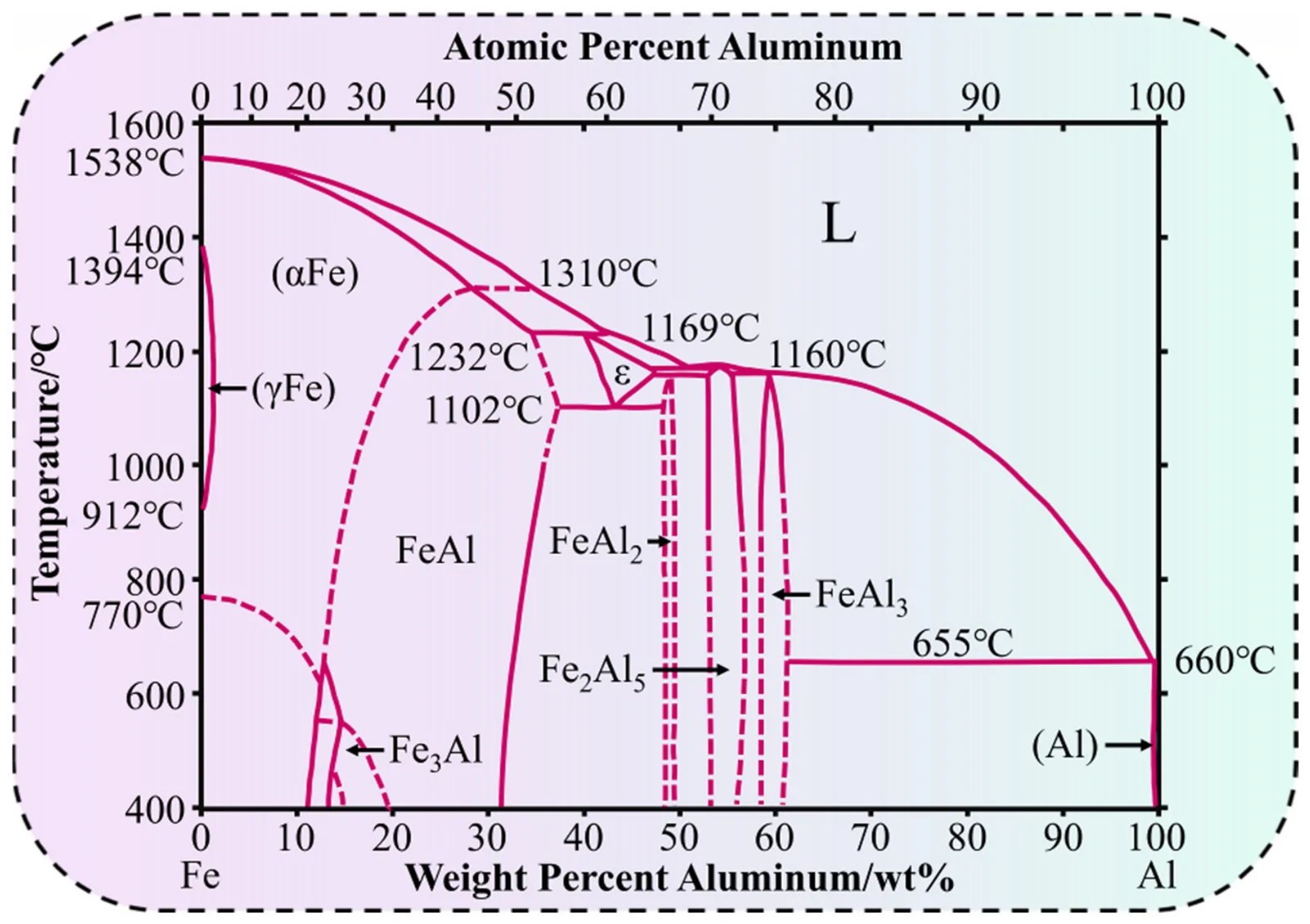
Fe-Al binary alloy phase diagram [22].

Al/steel composite structural joining has become a key method for achieving vehicle lightweighting. However, as shown in Table 1, significant differences exist in the thermophysical properties between Al and steel [11,12]. For instance, Al has a melting point of 660 °C, while that of Fe is about 1536 °C. During conventional welding processes, when Al reaches a molten state, steel has not yet begun to melt. Conversely, when steel has fully melted, the density difference between Al and steel causes liquid Al to stratify above the liquid steel [13,14]. This layering phenomenon leads to uneven distribution of joint composition, thereby compromising the joint mechanical performance. Therefore, precise control of heat input is critical for Al/steel dissimilar joining. Additionally, Al exhibits a linear expansion coefficient of about 24 × 10^−6^/°C and a thermal conductivity of about 238 W/(m·K), while those of Fe are 12 × 10^−6^/°C and 80 W/(m·K), approximately. The disparity in these properties causes significant thermal deformation and residual stress during the joints’ cooling and solidification phase. This leads to defects such as porosity and cracks within the joint, thereby degrading its performance [15]. Simultaneously, the surfaces of the Al alloys readily form an Al_2_O_3_ oxide film in the air. The oxidation reaction intensifies under high-temperature conditions. The surface oxide film impedes the joining between two liquid metals, reducing the joints’ reliability. Furthermore, as shown in the Fe-Al binary alloy phase diagram (Figure 1), Fe and Al are nearly completely immiscible at room temperature [16,17]. When the temperature increases to 655 °C, the solid solubility of Fe in Al remains at only 0.53%. Various factors contribute to the formation of Fe_x_Al_y_ intermetallic compounds (IMCs) at the Al/steel interface [18,19,20]. The contradiction between the hard–brittle characteristics and the load-bearing capacity of the IMC layer limits the mechanical performance of Al/steel joints.

**Figure 2:**
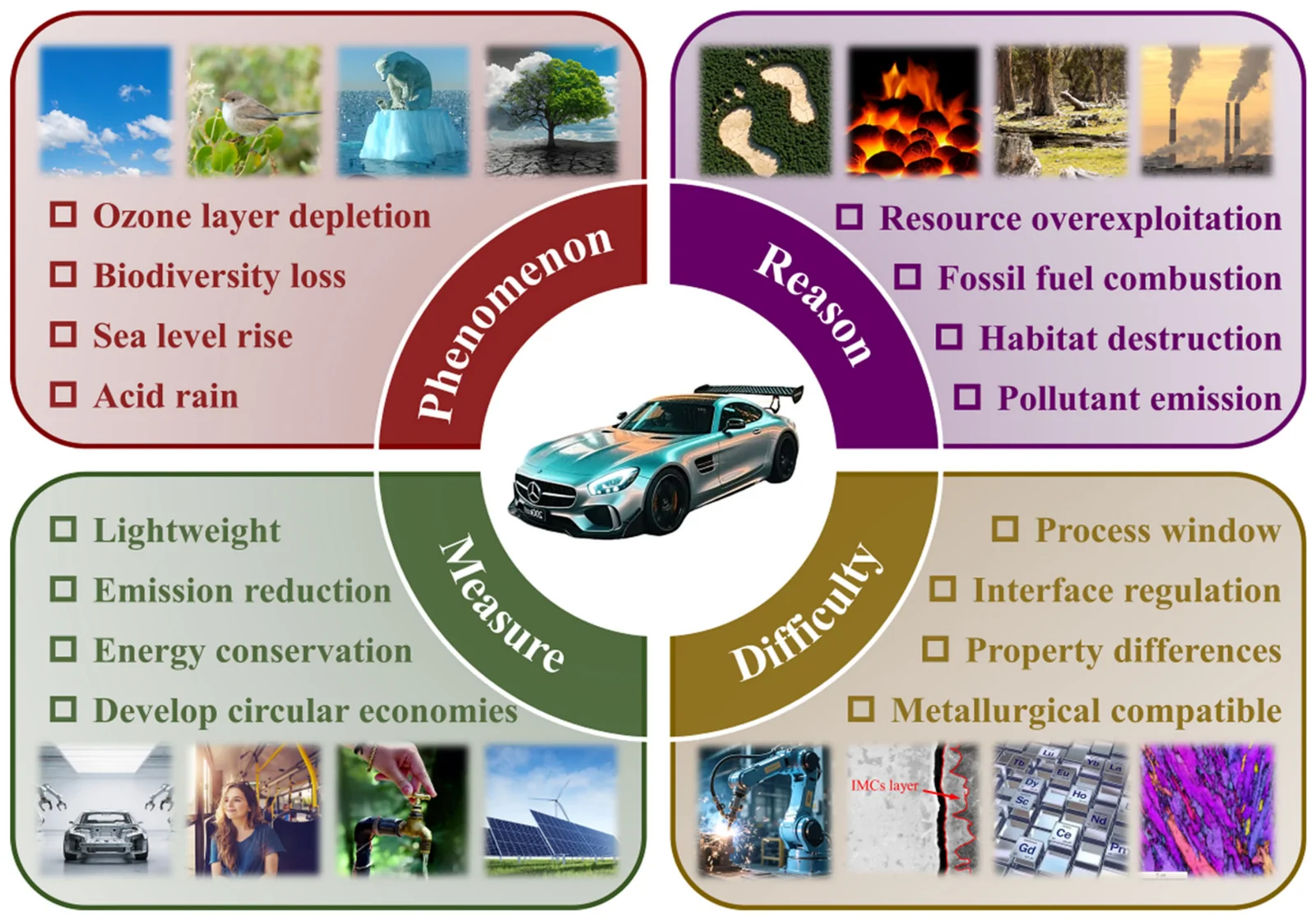
Overview of the research background, development trends, and industry pain points.

In conclusion, Figure 2 concisely illustrates the research background of this review, with numerous anthropogenic factors such as fossil fuel combustion leading to several global environmental issues. In addition, Figure 2 also elucidates that lightweighting has become a development and evolution trend in the relevant field, such as in automobile manufacturing. It highlights the key bottlenecks and core issues that the industry currently faces, thereby clarifying the practical significance and entry point of this review. In response to the aforementioned challenges, relevant scholars have conducted in-depth research and proposed a series of advanced Al/steel composite structural joining technologies. These processes aim to enhance weld performance by addressing the differences in thermophysical properties between Al and steel. They also seek to control the morphology, thickness, and distribution of the hard and brittle IMC layer [23]. This study categorizes Al/steel joining technologies into thermal joining (fusion welding, fusion brazing, solid-state welding, etc.), mechanical joining (clinching, self-piercing riveting, flow drill screw, etc.), and composite joining (such as resistance element welding, friction element welding, resistance rivet welding, etc.) [24]. This review examines Al/steel dissimilar joining across thermal, mechanical, and composite technologies, including their process variants. It establishes an integrated framework to explain how process parameters, fillers, interlayers, and external energy fields influence interfacial reactions and microstructural evolution, and how these changes affect defect formation, fracture behavior, and service reliability. Within this framework, the review critically assesses the mechanisms, advantages, and limitations of each joining approach, and compares their joint quality and mechanical performance, process complexity, technological maturity, and manufacturing applicability. By linking interfacial phenomena with process requirements and performance outcomes, this review identifies the factors and trade-offs that govern joint reliability. The resulting analysis provides a basis for selecting joining strategies for automotive applications and for identifying priorities for future research.

This study adopts a systematic review to examine dissimilar joining between Al alloys and steels. Relevant publications were identified through searches of Elsevier ScienceDirect using combinations of terms related to Al–steel joining, including thermal, mechanical, and composite processes. Studies were selected if they addressed Al/steel joining mechanisms, process variants, interfacial reactions or microstructural evolution, defect formation, fracture or mechanical performance, durability, or manufacturing applicability. Peer-reviewed research articles and relevant review papers were considered, with emphasis on recent advances while retaining earlier studies that established key mechanisms or joining approaches. Studies on other material combinations were included only when they provided directly relevant mechanistic or methodological insight. The selected literature was synthesized qualitatively and comparatively; quantitative ranking was avoided where differences in materials, joint configurations, and test conditions prevented direct comparison.

## 2. Thermal Joining Technology

### 2.1. Fusion Welding

Fusion welding refers to the process of heating the base material (BM) to a molten state, where the molten plates undergo elemental diffusion and liquid mutual solubility without applying pressure. The molten pool recrystallizes as the temperature decreases, ultimately forming the welded joint [25]. However, it is challenging for traditional arc fusion welding to regulate the energy density. Low energy density results in a large heat-affected zone (HAZ), allowing extensive reaction between Fe and Al elements and forming a thick hard–brittle IMC layer at the interface [26]. Conversely, high energy density can cause defects such as porosity and cracks, significantly degrading the mechanical performance of the joint. Researchers in related fields have employed advanced welding technologies and optimized process parameters to control the morphology and thickness of the IMC layer at the Al/steel interface [27,28]. These efforts aim to continuously improve joining processes and enhance joint performance.

**Figure 3:**
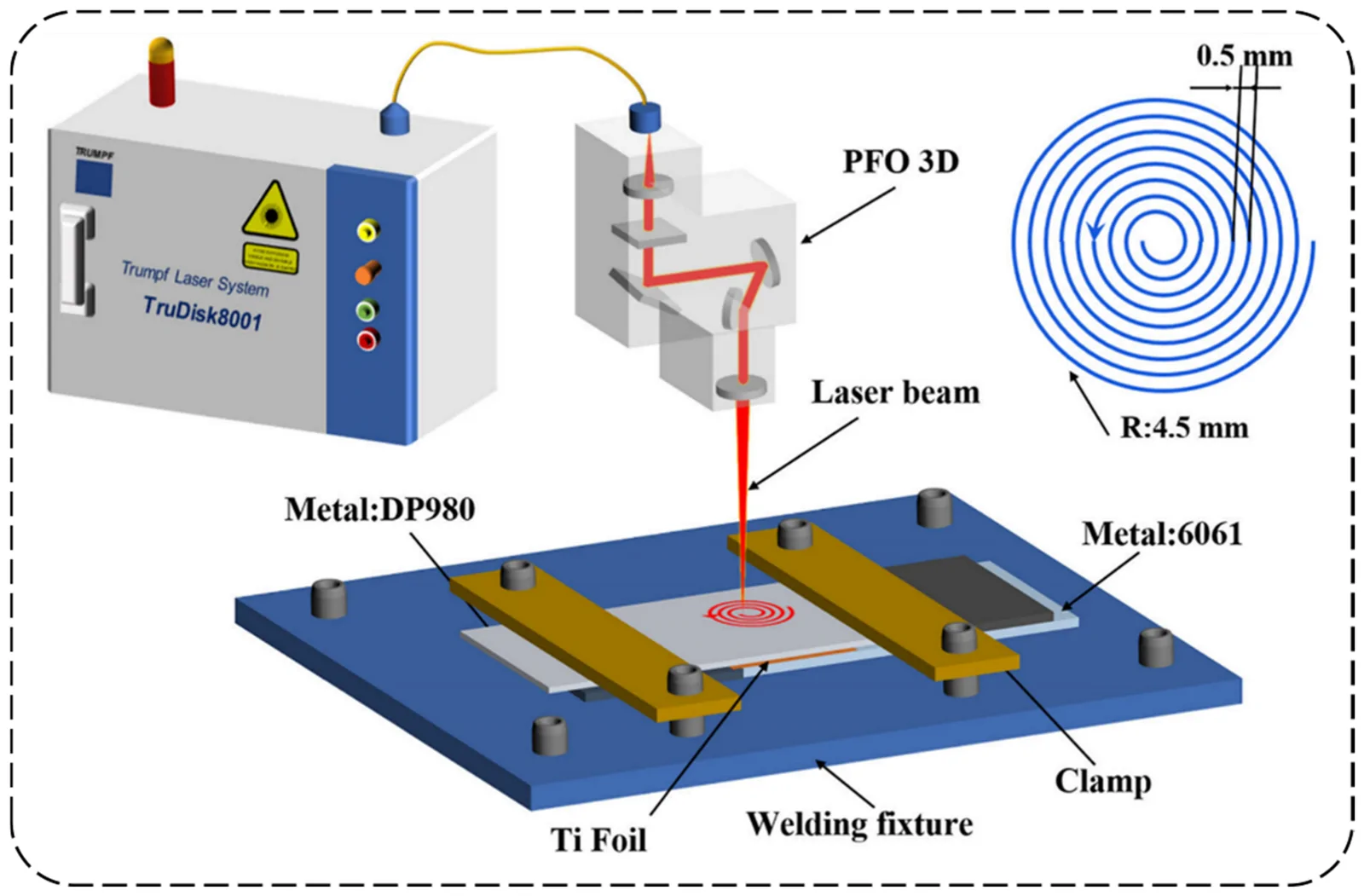
Schematic of laser spiral fusion welding setup [29].

**Figure 4:**
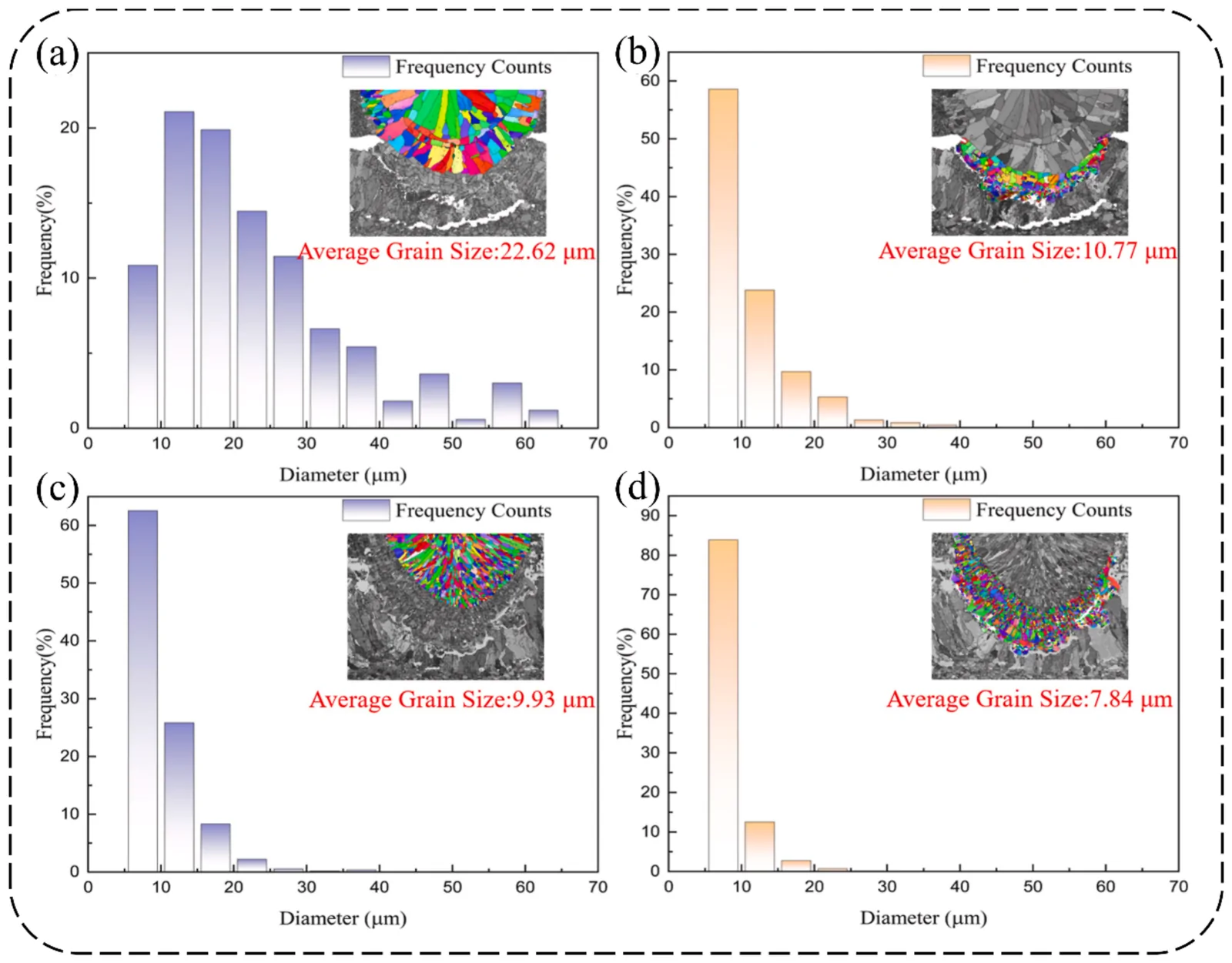
Grain size distribution of joints: (**a**,**b**) without interlayer and (**c**,**d**) with Ti interlayer [29].

To address the conflict between excessive growth of hard and brittle IMCs and maintain load-bearing capacity, researchers have adopted several strategies: adjusting process parameters, incorporating intermediate transition layers [29,30], and applying external energy fields [31]. These strategies aim to improve metallurgical bonding quality and structural compatibility at the interface. The Ti interlayer in laser spiral fusion welding (LSFW) (Figure 3) acts as a multifunctional transition layer rather than merely an additional constituent at the Al/Fe interface [29]. The results indicate that the Ti interlayer effectively enhances laser absorption and increases penetration depth. Although the flow pattern within the molten pool remains unchanged, the uniformly dispersed TiO particles promote grain refinement, reducing the average grain size in the upper weld region and the solute band by 56.1% and 27.3% (Figure 4), respectively. According to the Hall–Petch theory, grain refinement simultaneously enhances the yield strength and impact toughness of the joint. Moreover, the TiO particles suppress the formation of the Fe_2_Al_5_ and Fe_4_Al_13_ phases, leading to a 27.86% increase in shear strength and a 15.06% increase in peel strength of the joints.

Ni transition layers provide a distinct strategy by modifying the interfacial reaction pathway rather than simply suppressing intermetallic formation. Yu et al. [30] introduced Ni to replace the direct Fe–Al reaction with a Ni–Al reaction in laser-arc hybrid welding (LAHW). At low heat input, a brazing-like interface forms between liquid Al and solid Ni, while increasing heat input induces local melting and promotes the formation of AlNi, Al_3_Ni_2_, and Al_3_Ni. The resulting 87.5% increase in ultimate tensile strength demonstrates that redirecting the reaction pathway can substantially improve joint performance. The significance of the Ni interlayer lies in its ability to decouple the direct Fe–Al reaction and provide a more controllable interfacial reaction route. However, replacing Fe–Al IMCs with Ni–Al phases does not inherently eliminate brittleness. The benefit of this strategy depends on the thickness, continuity, distribution, and fracture resistance of the newly formed Ni–Al phases. Thus, the novelty of Ni interlayer design is not simply the reduction in Fe–Al IMCs, but the deliberate engineering of the interfacial reaction pathway to obtain a more favorable phase constitution and mechanical response. This also highlights that interlayer effectiveness should be evaluated based on the nature and mechanical integrity of the resulting interfacial phases, rather than solely on the amount of Fe–Al IMCs suppressed.

Li et al. [31] employed magnetic-assisted laser beam welding (MA-LBW) to join Al/steel dissimilar materials. The results demonstrate that the MA-LBW joint exhibits increases of 22.22% in ultimate tensile load and 45.76% in elongation compared to conventional laser welding without a magnetic field. These gains should be interpreted as study-specific improvements over the unassisted control condition. They do not, by themselves, establish that MA-LBW outperforms an optimized conventional laser-welding process unless the baseline was independently optimized using the same material system, joint configuration, and test conditions. The effect of the additional magnetic field is to break the continuous brittle IMC layer into dispersed particles and optimize the phase structure, but it does not fundamentally eliminate the formation of IMCs. Instead, the magnetic field non-contactly drives the flow of liquid metal, ensures uniform distribution of IMCs at the interface, suppresses component segregation, and reduces the tendency to weld cracking.

### 2.2. Fusion Brazing

Traditional fusion welding of Al/steel dissimilar materials tends to form excessively thick IMC layers at the interface due to intense atomic interdiffusion, which compromises joint reliability. To overcome the limitation, fusion brazing has been developed by integrating principles of fusion welding and brazing [32]. This hybrid approach leverages the difference in melting points between Al and steel to precisely control welding heat input, melting the low-melting-point Al alloy BM or Al-based filler metal while keeping the high-melting-point steel BM in a solid state. A fusion weld joint is formed on the Al side; simultaneously, the molten material wets and spreads over the solid steel surface, creating a brazing joint [33]. Consequently, a thin and continuous IMC layer (predominantly composed of Fe_2_Al_5_/FeAl_3_) develops at the interface. In contrast to the conventional fusion welding, fusion brazing restricts the IMC layer to micrometer or even submicrometer thickness [34]. This not only ensures sound metallurgical bonding but also alleviates stress concentration induced by mismatch in thermal expansion coefficient and elastic modulus, thereby effectively inhibiting brittle fracture of the joint.

**Figure 5:**
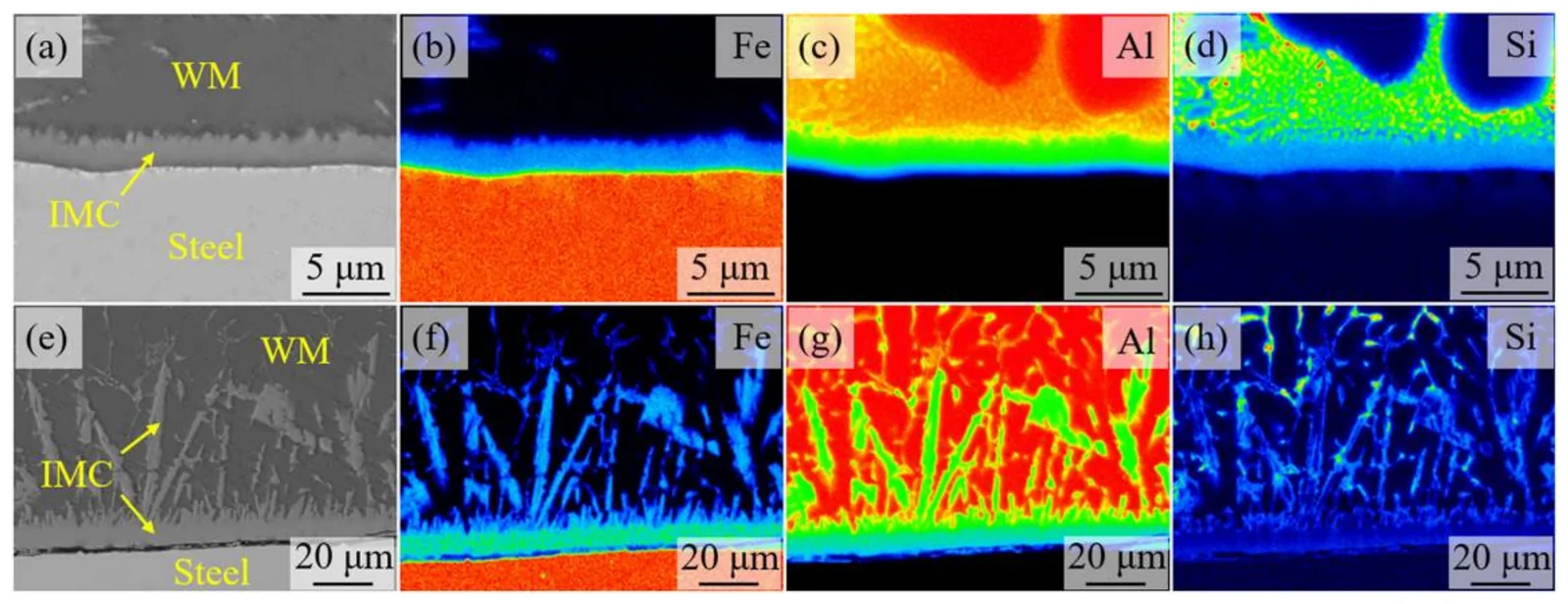
The SEM and EDS images of the interfacial area of joints prepared using ER4047 filler metal: (**a**–**d**) laser tilt angle: 10°; (**e**–**h**) laser tilt angle: 20° [36].

Fusion brazing technology offers distinct advantages in controlling energy input and suppressing IMC growth. Yang et al. [35] found that Si participates in interfacial reactions and reduces IMC formation. First-principles calculations further suggest that Si can substitute Al atoms in Fe-Al IMCs and form Fe-Al-Si phases with lower brittleness. Li et al. [36] compared ER4047 and ER5356 filler metals and showed that ER4047 forms a ternary Fe-Al-Si IMC layer, while ER5356 produces a binary Fe-Al layer (Figure 5). Under the same welding conditions, ER4047 produces a thinner IMC layer. These results are consistent in showing that Si can suppress IMC growth and alter phase constitution. The advantage of Si-containing fillers lies in their ability to simultaneously modify interfacial chemistry and wetting behavior, thereby reducing and chemically modifying Fe–Al reaction products. However, their effectiveness cannot be judged solely by IMC thickness, because Si also changes weld-metal solidification and the resulting fracture path.

**Figure 6:**
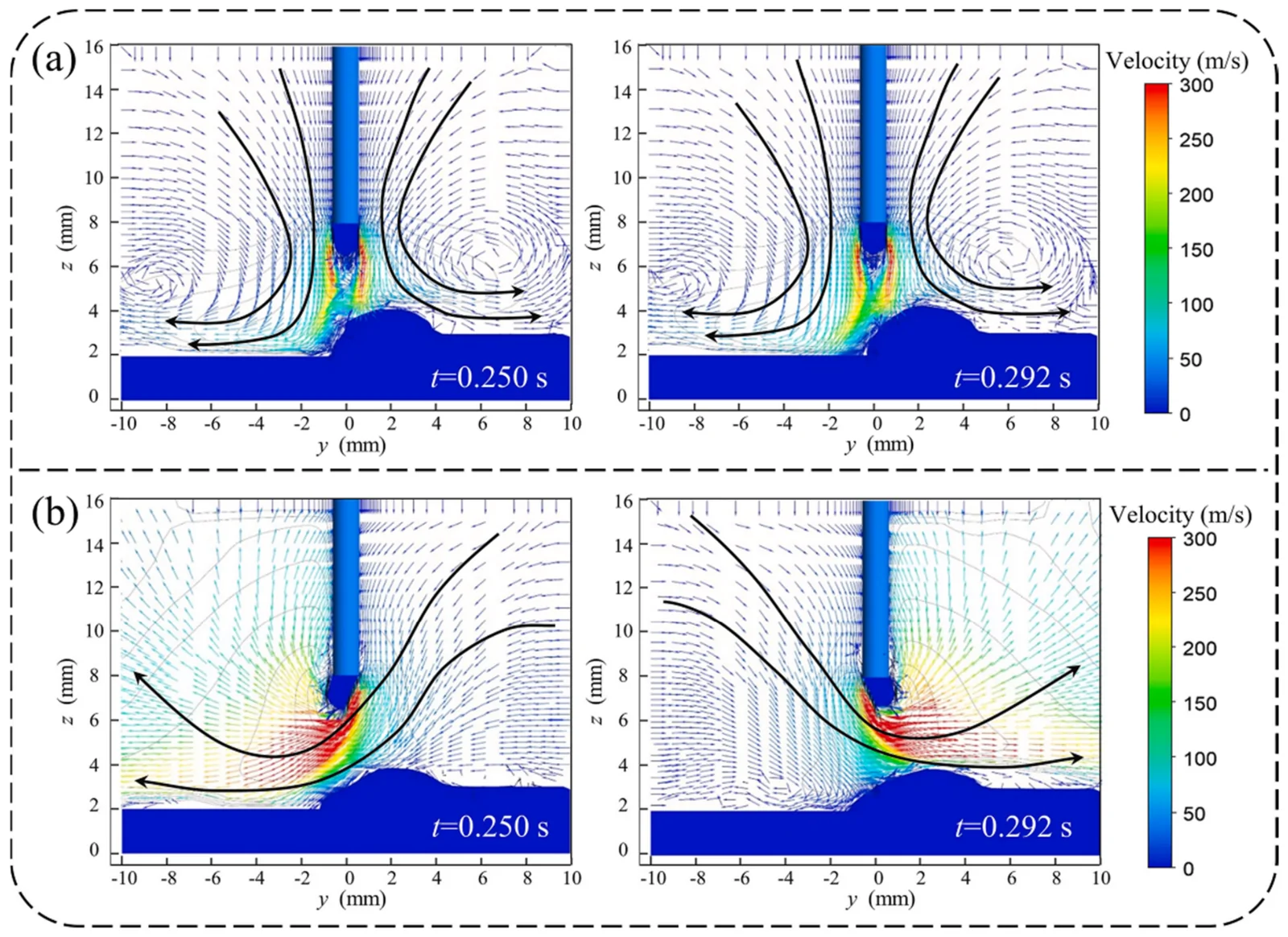
Effect of the LEMF on the arc plasma velocity: (**a**) without LEMF and (**b**) with LEMF [37].

Magnetic-field-assisted MIG fusion brazing demonstrates that thermal effects and flow behavior cannot be considered independently in Al/steel joining. Wang et al. [37] developed a three-dimensional arc plasma–liquid Al–solid steel coupling model and showed that an alternating longitudinal external magnetic field induces periodic arc oscillation, modifies droplet transfer, and promotes lateral spreading of molten Al (Figure 6). Notably, the magnetic field increases the molten-pool width while decreasing its peak surface temperature, indicating that enhanced spreading does not necessarily require higher heat input. Instead, the redistribution of arc force, droplet momentum, and electromagnetic force provides an alternative pathway for controlling molten-metal transport. This is particularly important for Al/steel joining, where sufficient spreading is required for effective wetting, whereas excessive thermal input promotes Fe–Al IMC growth. By improving spreading while limiting localized overheating, magnetic-field assistance can potentially decouple wetting control from excessive heat input and thereby expand the processing window for joint formation. The significance of this approach therefore lies not simply in electromagnetic stirring, but in using an external force field to simultaneously regulate heat and mass transfer, with consequent effects on interfacial reactions and joint quality. The key significance of magnetic-field assistance is therefore its ability to decouple molten-metal spreading from excessive heat input, providing a new process-control pathway for balancing wetting, thermal exposure, and interfacial reaction in Al/steel joining.

Resistance spot welding (RSW) utilizes opposing electrodes to apply pressure and pass current through the workpiece. The resulting resistive heat generates localized melting at the contact surfaces. The molten nugget solidifies after current cessation, ultimately forming a metallurgical joining [38]. This process is fundamentally based on “localized selective melting”. The significant differences between Al/steel in thermophysical properties are leveraged. By precisely controlling welding current, time, and electrode pressure, the low melting point of Al allows for localized micro-melting, while steel remains largely solid due to its high melting point. The molten Al interacts with the solid steel surface, promoting rapid liquid–solid diffusion and the formation of an ultrathin (typically 1~5 μm) and continuous IMC layer [39]. The RSW process achieves metallurgical joining while effectively restraining brittleness caused by excessive IMC growth. Ding et al. [40] proposed a linear actuating enhanced resistance spot welding (LAE-RSW) process. This technique embeds a linear actuator capable of generating 200 Hz pulsed driving forces within the positive electrode, superimposing pulsed driving forces onto the static electrode pressure during the welding cycle [41]. It promotes melt flow within the weld nugget by dynamically adjusting electrode pressure, thereby altering the formation process of IMC layers. The linear actuator alters the temperature distribution within the weld nugget, promoting heat transfer from the center toward the periphery. It results in increases of 10.5% and 54.9% in the nugget diameter and cross-sectional area, respectively [42]. Piezoelectric vibration induces temperature reduction at the Al/steel interface and fragmentation of IMC layer tips, simultaneously reducing peak IMC layer thickness by 81.9% and increasing the joint’s average peak tensile-shear load by 38.5%.

The oxide layer on the surface of Al alloys is another bottleneck specific to resistance welding. Multi-ring domed (MRD) electrodes deform the Al alloy surfaces and break the insulating oxide film, improving current transfer and welding stability [43,44]. Shi et al. [45,46] showed that AA6022/high-strength low-alloy steel (HSLAS) and AA6022/low carbon steel (LCS) joints can exhibit similar IMC strength but different tensile-shear performance. This difference indicates that local interfacial strength is not always the controlling factor for joint load capacity. Sheet strength, nugget geometry, stress concentration, and lap type can change the measured failure load even when the IMC layer appears comparable.

Hot cracking in the Al-side of the nuggets adds another layer of complexity. Li et al. [47,48] adapted Rappaz–Drezet–Gremaud (RDG) and Suyitno-Kool–Katgerman (SKK) criteria to RSW and compared AA7075 and AA6061-T6. The results indicate that AA7075 has higher hot crack susceptibility, while AA6061 is less sensitive. The refined RDG criterion better matches the overall experimental results, whereas SKK performs more accurately in the equiaxed grain region. This difference between criteria is not merely a modeling issue; it reflects the spatial heterogeneity of the nugget solidification microstructure. Longitudinal temperature gradient and grain size are identified as controlling factors, which means that alloy composition, thermal gradient, and solidification morphology jointly determine cracking susceptibility.

Newton Ring electrodes further highlight the importance of solidification path control [49,50]. By promoting controlled outward-to-inward solidification, it suppresses spatter and reduces sheet deformation. This strategy differs from MRD electrodes, which mainly improve oxide disruption and current input stability. The two electrode concepts target different stages of welding: one acts during current transfer and surface breakdown, while the other modifies molten nugget solidification.

### 2.3. Solid-State Welding

Solid-state welding joins Al and steel below their melting points through pressure, friction, vibration, or impact, and joining occurs through solid-state diffusion, eutectic diffusion, plastic deformation, or mechanical interlocking [51,52]. Its main advantage is the reduction in an excessive Fe-Al reaction. However, several studies show that IMCs can still form in friction-based joining and can continue to grow during service exposure. The difference from fusion welding lies in the reduced thickness, discontinuity, and slower growth kinetics of the reaction layer, rather than complete absence of interfacial phases.

Friction stir welding (FSW) creates a thermo-mechanically affected interface through the rotation and translation of a non-consumable tool [53]. The joint strength originates from a combination of mechanical interlocking, grain refinement, plastic mixing, and diffusion bonding. The welding temperature is usually controlled below the range where violent Al/steel reactions occur, often around 600~650 °C [54]. It makes FSW suitable for suppressing thick brittle Fe-Al IMCs, while the severe plastic deformation helps disrupt surface oxides and enlarge real contact area. Moreover, as a solid-state joining method, it requires no filler materials or shielding gases and produces no fumes, arc light, or spatter. Its limitation is that the joint quality is highly sensitive to tool geometry, plunge depth, offset, material placement, and local heat generation.

**Figure 7:**
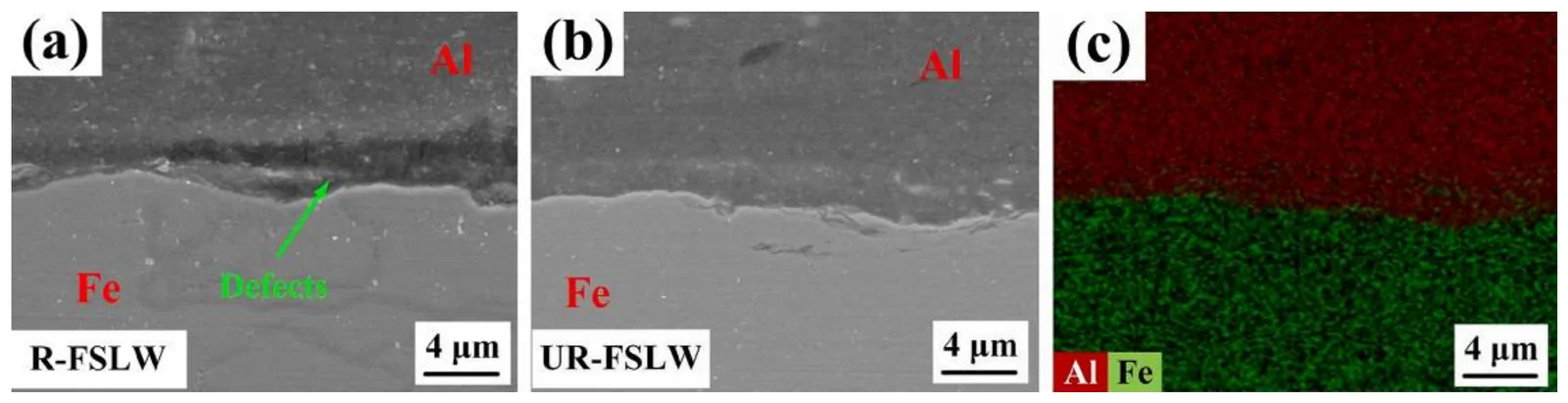
Microscopic morphology of the bonding interface and grain evolutionary characteristics: (**a**) R-FSLW, (**b**,**c**) UR-FSLW [55].

Hybrid FSW variant techniques attempt to overcome these problems by adding vibration or self-riveting. Yin et al. [55] applied ultrasonic vibration and self-riveting to friction stir lap welding (FSLW) of 6061-T6 Al alloy and Q235 steel. Compared with regular FSLW, the UR-FSLW joint (Figure 7a) showed a denser interface with fewer crack-like defects (Figure 7b,c). This result indicates that ultrasonic vibration promotes material flow and defect closure, yet the mechanism is not limited to acoustic softening. Service reliability studies reveal a contradiction often overlooked in process-focused research. Wang et al. [56,57] investigated continuous drive friction welded 1060 Al/Q235 joints under coupled thermal, electrical, and mechanical loading. After 60 days, the IMC layer thickness in the 1/2R region increased from 1.25 μm to 2.39 μm, while the Al/Fe interdiffusion distance also increased. FeAl, FeAl_2_, and Fe_2_Al_5_ phases were identified, and the interfacial evolution accelerated failure. This result challenges the common assumption that a thin IMC layer guarantees long-term stability. Even when solid-state welding produces a favorable initial interface, service temperature, current density, and static load can reactivate diffusion and cause delayed embrittlement.

To investigate the formation and evolution of the IMC layer, Wu et al. [58] employed FSW and ultrasonic vibration-enhanced friction stir welding (UVeFSW) to achieve welding of Al/steel dissimilar materials. The results indicated that ultrasonic vibration significantly refined the microstructure and reduced IMC growth. Specifically, the average grain diameter of the Al alloy decreased by 20.57% in the central zone and 15.58% in the interface zone. The proportion of recrystallized grains increased from 0.99% and 57.37% to 13.27% and 59.62%, respectively. Moreover, the average IMC thickness decreased from 2.71 μm in conventional FSW to 1.22 μm in UVeFSW [59]. Ultrasonic vibration enhanced material plastic flow, particularly along the weld depth direction, and reduced the peak welding temperature by 5.13% on the Al side and 4.88% on the steel side, thereby suppressing excessive IMC formation.

**Figure 8:**
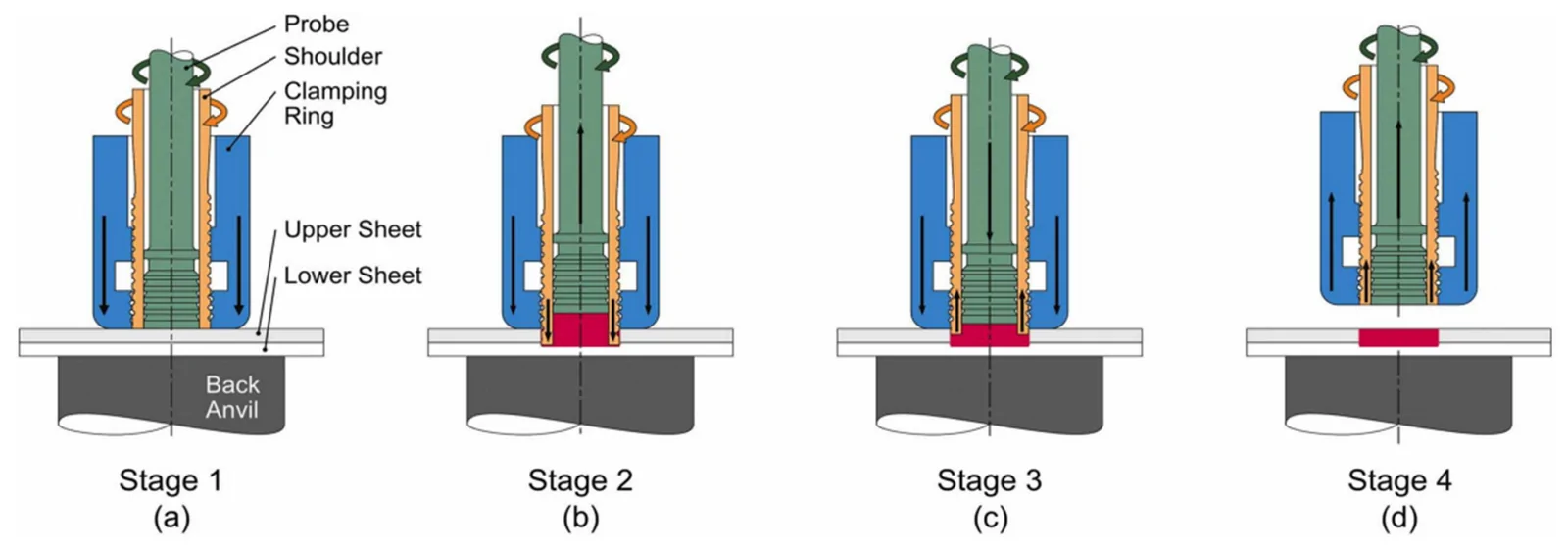
Four stages of the RFSSW process: (**a**) clamping stage, (**b**) plunging stage, (**c**) refill stage, (**d**) retraction of the tools [61].

However, conventional friction stir spot welding (FSSW) often leaves keyholes at the weld center, creating stress concentration points that compromise surface appearance and fatigue performance [60]. In contrast, refill friction stir spot welding (RFSSW) utilizes the relative motion between the sleeve and stir pin to re-extrude thermoplastic material generated during friction into the weld region. As shown in Figure 8, rotation ceases after the joint is smoothed, and welding is achieved upon metal solidification [61]. The RFSSW process eliminates the keyhole defect inherent in conventional FSSW, thereby increasing the effective load-bearing area of the joint and enhancing its mechanical performance.

Explosive welding (EW) utilizes a high-speed, high-pressure shock wave from a controlled detonation to induce hydrodynamic instability at the Al/steel interface. The EW process generates a characteristic wavy bonding morphology, with wavelengths typically ranging from tens to hundreds of micrometers [62]. The wave peaks and troughs create a three-dimensional mechanical interlock, significantly enhancing joint strength. Furthermore, although the interface temperature can transiently reach the melting point, the extremely short duration (on the microsecond scale) of the thermal pulse severely restricts atomic diffusion [63]. As a result, only an ultrathin (typically <1 μm) and discontinuous intermetallic compound layer forms along the wave crests, preventing the development of extensive brittle phases. Moreover, as a solid-state welding process without external heat input, the BM will not undergo grain growth or phase transformation, thereby retaining its original mechanical properties. Nevertheless, it is worth noting that the explosion shock can generate significant residual stress within the material, affecting the dimensional stability and service life of the component.

**Figure 9:**
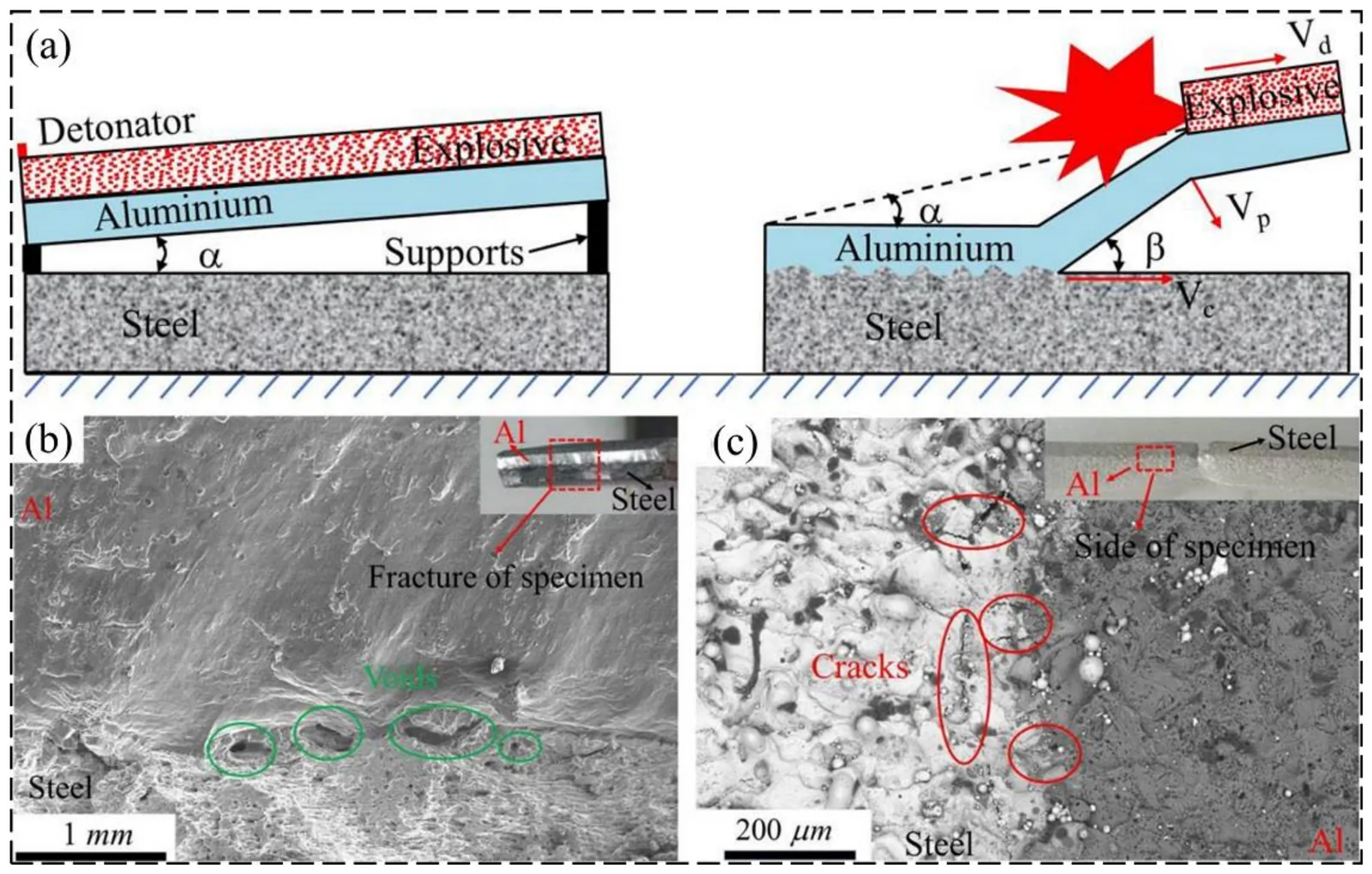
Characteristics of the explosion welded interface: (**a**) schematic of EW, (**b**) interface characteristics of tensile specimen fracture, and (**c**) microcracks on the side of the tensile specimen [64].

Huang et al. [64] found that conventional damage fracture theories cannot predict the elongation of explosion-welded Al/steel composite plates. First, uniaxial tensile tests and scanning electron microscopy (SEM) tests were used to analyze the deformation behavior and interfacial microstructure of the explosive welded Al/steel composite plate (Figure 9a,b). Results revealed wave structures, brittle phases, and microcracks in the composite plate interface layer (Figure 9c). The uniform elongation and total elongation of the Al/steel composite plate deviated significantly from the linear hybrid law. Second, a simulation model was developed to analyze the effects of interface wave structures, brittle phases, and interfacial microcracks on the instability and fracture behavior of explosion-welded Al/steel composite plates [65]. Interface wave structures enhance the elongation of the composite plate, while the uniform elongation decreases with the increase in brittle phases and microcracks.

On the contrary, the EW process also has significant limitations. First, it requires strict establishment of safety isolation zones. Second, it is typically only suitable for joining thick sheets. In contrast, ultrasonic welding (UW) achieves high-quality joining by inducing plastic deformation in the joint microzone through high-frequency mechanical vibration (typically 20~60 kHz) [66]. The UW process is generally suitable for thin sheets less than 3 mm thick, with a temperature rise in the weld zone of only 200~400 °C. Consequently, it completely avoids over-aging of Al alloys and phase transformations of steel, preserving the original performance of the BM [67]. Characterized by micro-scale, low-temperature, high precision, and controllable nature, UW is the preferred method for reliable low-temperature joining of dissimilar materials in microelectronics, new-energy batteries, and precision instruments. Bajaj et al. [68] employed ultrasonic spot welding (USW) to fabricate joints between AA2024-T3 Al alloys and galvanized HSLA steel. An Al-Zn diffusion layer (approximately 30~70 μm) forms at the weld interface, with no brittle IMCs detected. The average tensile lap shear strength is about 169 MPa. Compared to the welding energy of 1000 J, the higher welding heat input at 3000 J results in a thicker and more uniform Al-Zn diffusion layer. In summary, the strengths of Al/steel dissimilar joints obtained through thermal joining techniques are listed in Table 2.

Across fusion welding, fusion brazing, and solid-state welding, Al/steel joining faces an interfacial dilemma. The joint must develop sufficient joining to transfer load while limiting Fe–Al reactions to prevent a continuous brittle IMC layer from controlling fracture. Each process resolves only part of the dilemma. Fusion welding provides metallurgical continuity and flexible seam formation, yet molten pool instability, porosity, cracking, and thick Fe-Al IMCs remain difficult to avoid. Fusion brazing narrows the reaction zone by keeping steel solid, although joint quality depends on wetting, filler chemistry, and interface continuity. Solid-state welding reduces heat input and usually suppresses thick IMCs; incomplete bonding, geometric defects, and residual stress limit reliability.

## 3. Mechanical Joining Technology

### 3.1. Clinching

Mechanical joining provides a different solution to Al/steel dissimilar joining because the joint is formed mainly through plastic deformation, geometric constraint, and mechanical interlocking rather than metallurgical reaction. Compared with thermal joining technologies, its joint strength is often lower, but it avoids heat input, filler materials, shielding gas, and Fe-Al IMC formation. It makes mechanical joining attractive for low to medium load sheet structures, especially where cost, cycle time, and thermal distortion are critical. However, the absence of IMCs does not mean that the joint is free from interfacial reliability problems. The main failure source shifts from brittle reaction layers to insufficient interlocking, local cracking, corrosion of the Al sheets, fatigue damage around the deformed region, and geometric defects produced during forming.

Clinching is the typical representative process. It joins sheets by forcing them between a punch and die, where local plastic deformation produces an undercut and forms a mechanical interlock [69]. Additionally, work hardening occurs in the deformed zone, slightly enhancing strength while the surrounding base material retains its original microstructure and performance with no HAZ or softened band formed. To further optimize joining effectiveness, grooves are typically incorporated into the die or punch. These grooves accelerate material flow, facilitating easier interlocking. Liao et al. [70] joined the Al/steel structure via electromagnetic-assisted clinching (EAC) for the first time. The joints were exposed to the neutral salt spray environment, and mechanical degradation laws and failure mode evolution were obtained. The peak shear and pull-out load of joints decreased by 22.6% and 41.5% at the aging time of 24 days, respectively. The pull-out performance of joints was greatly affected by salt spray aging, compared with the shear performance [71]. Moreover, the degradation of shear performance was mainly due to the gradual corrosion and dissolution of the Al sheet under salt spray environment, especially the neck of the Al sheet. However, the degradation of the pull-out performance was mainly due to the damage of the interlocking structure. Therefore, it was necessary to develop appropriate corrosion protection measures for Al/steel joints, such as adopting EAC-adhesive composite joints or plating for the sheets [72].

Clinching relies on plastic deformation of sheet metal to achieve joining, which can cause cracking in HSS with limited ductility. Therefore, Busse et al. [73] developed shear-clinching (S-C) by combining clinching with shear cutting. As a single-step process, it does not require additional fasteners or heat input. This technique demands good toughness in the punch-side plate, while the lower-ductility material on the die side requires pre-formed holes. The punch forces material into these preformed holes, forming an interference fit joint. The die depth for the upper sheet is reduced to prevent deformation concentration at the punch corners [74]. For the lower sheet, the die groove is omitted to minimize tensile stress in the HSS, thereby significantly lowering the difficulty of joining low-ductility materials.

### 3.2. Self-Piercing Riveting

Self-piercing riveting (SPR) is more mature and widely adopted in automotive lightweight structures. Compared with clinching, SPR usually provides higher joint strength and better fatigue resistance, and it eliminates pre-punching [75]. The semi-tubular rivet pierces the upper sheet and flares into the lower sheet under die constraint, producing a strong mechanical interlock. Its industrial value lies in automation compatibility and tolerance to dissimilar material joining. Nevertheless, SPR transfers the joining challenge to rivet deformation, sheet cracking, bottom thickness, and die-side protrusion. For Al/steel joints, the material arrangement and the ductility of the lower sheet strongly determine whether a stable interlock can be formed.

**Figure 10:**
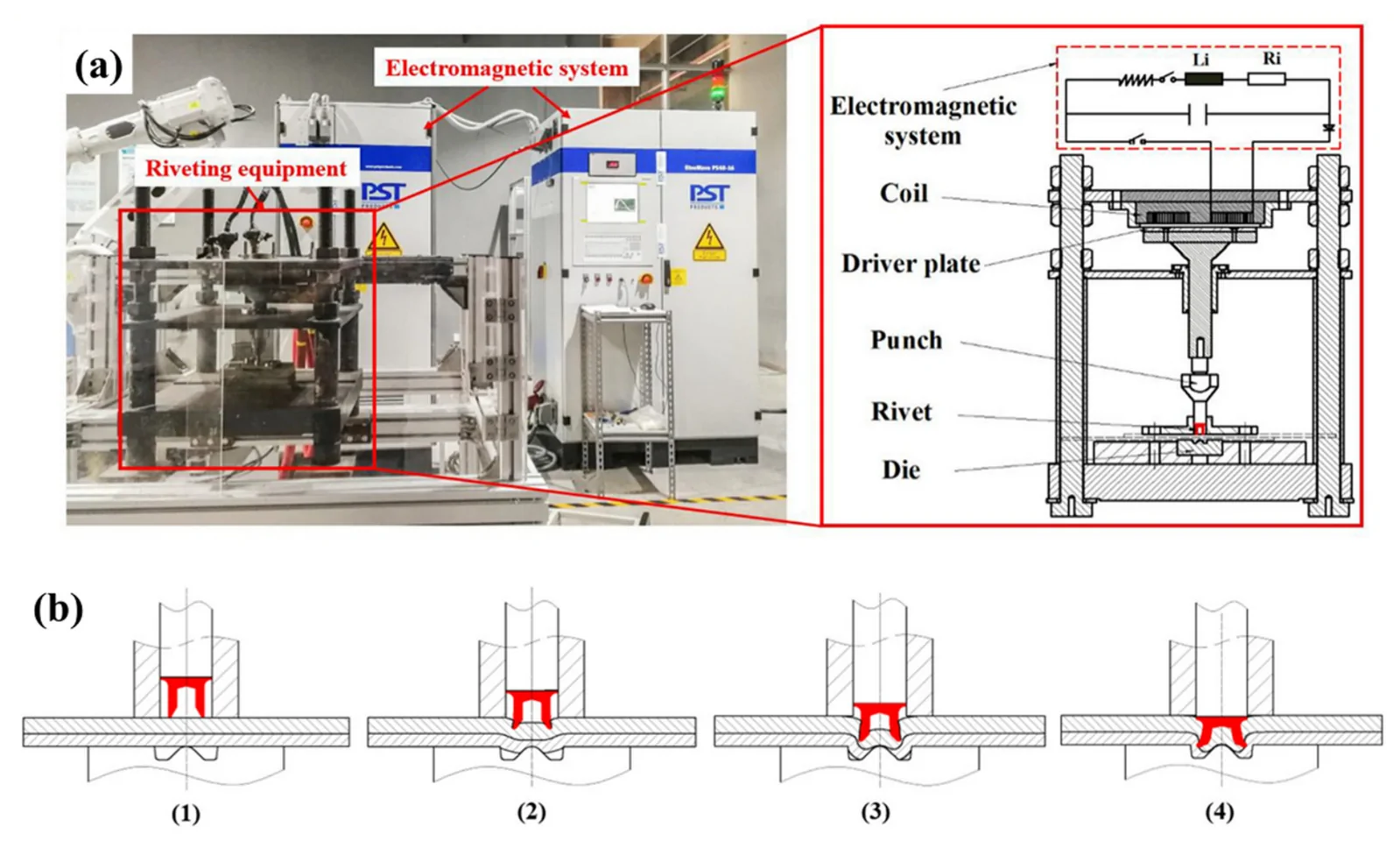
Electromagnetic self-piercing riveting: (**a**) riveting equipment and (**b**) riveting process [76].

Several SPR variants target this problem from different directions. Jiang et al. [76,77] examined electromagnetic SPR (E-SPR) and found that rivet dimensions strongly affect the mechanical response of Al/steel joints (Figure 10). When the rivet pierces the upper sheet UHSS, upsetting, bending, and fracture may occur. Increasing the rivet leg tip angle reduces the average undercut, with the smallest undercut observed at 40°. This indicates that a rivet geometry suitable for ductile Al may not work for UHSS, where penetration resistance and rivet deformation become dominant. Thermally assisted SPR (TA-SPR) uses local induction heating to improve UHSS ductility before riveting [78]. This method involves locally preheating the steel with an induction coil prior to conventional SPR. Induction heating enhances the ductility of UHSS sheets while reducing its deformation resistance, thereby improving the riveting ability of Al alloy components to UHSS sheets.

Conventional self-piercing riveting (C-SPR) joints tend to fail on the weaker steel sheet side. To enhance the fatigue life and mechanical performance of SPR joints made from thin sheet steel, Liu et al. [79] proposed a gasket-assisted self-piercing riveting (GA-SPR) process and investigated the effects of washer thickness and diameter on joint performance. The main conclusions can be summarized below: compared with C-SPR joints, GA-SPR joints showed higher undercut values (average increase of 16.4%) and the bottom thickness increased by 1.6 times. Both undercut values and bottom thickness decreased as washer thickness increased [80]. The peak force of GA-SPR joints was generally higher than that of C-SPR joints. Specifically, the G06-25 joint exhibited a 30.71% increase in peak force and a 97.04% increase in energy absorption. However, increases in washer thickness reduced both the peak force and energy absorption of GA-SPR joints. While larger washer diameters produced only a modest rise in peak force, they significantly increased the failure displacement, leading to greater energy absorption [81]. Notably, an excessively thin washer diminishes the improvement in joint performance during failure.

To mitigate bottom protrusion of joint and improve joining strength, Wu et al. [82] introduced a reshaping self-piercing riveting (R-SPR) process. The stress–strain distribution and forming characteristics during reshaping were analyzed, and the mechanical performance as well as failure mechanisms of SPR and R-SPR joints were compared. The results indicate that reshaping process significantly reduces bottom protrusion height and promotes secondary rivet flaring. After reshaping, the maximum shear strength increased by 20% for the pip die and 28% for the flat die, while peel strength improved by 24% and 25%, respectively [83]. The flat die demonstrated superior reshaping performance.

The innovation of integrated die-casting technology has created unprecedented opportunities for automotive lightweighting [84]. This approach enables the direct formation of components in a single die-casting process while the material is in a molten state, thereby eliminating many conventional manufacturing steps such as stamping and welding between components [85]. It simplifies production processes, reduces part counts, and fulfills multiple demands—cost savings, lower energy consumption, and extended driving range—achieving efficient conversion from raw materials to finished products. However, the die-casting Al alloys typically exhibit lower ductility. Experimental results indicate that materials with lower ductility are prone to cracking during the SPR process, which critically impacts joint quality and reliability. Zhao et al. [86] establishes a two-dimensional finite element model to investigate the cracking mechanism of NHT die-casting Al alloys when riveted to steel sheets. This study concludes through simulation combined with experimentation that for bottom sheet made of NHT die-cast Al alloys, the following strategies are recommended to reduce cracking risk [87]. First, minimize rivet depth to decrease plastic flow strain in the bottom sheet and promote an earlier transition to a compressive stress state. Second, increase die fillet radius or adopt spherical die to achieve more uniform stress distribution across the bottom sheet and reduce localized plastic strain.

### 3.3. High-Speed Joining

High-speed joining (RIVTAC^®^) is a novel mechanical joining process [88] that requires no pre-drilled holes. It achieves joining by driving long rivets into the BM through high-speed stamping of auxiliary components. Part of the top layer material is extruded upward into the lower cavity of the rivet head, ensuring the strength of the joint [89]. A critical requirement for this technology is that the sheets have sufficient stiffness and strength to withstand rivet penetration without excessive deformation.

Meschut et al. [90] achieved joint strengths of up to 6.86 kN by high-speed impact joining 2 mm thick AA6061 Al alloy sheets to 2 mm thick 22MnB5 PHS sheets. High-speed joining technology can also utilize explosive-driven nails to penetrate sheets, thereby achieving reliable joining. Gao et al. [91] investigated the effects of nail head geometry and target sheet structure and strength on penetration depth through numerical simulation. Under identical explosive propulsion forces, cone-head nails and flat-head nails exhibited larger penetration depths, whereas hollow nails underwent severe plastic deformation and achieved smaller penetration depths. Furthermore, as the cone angle of the nail head increased, the effective penetration depth first rose and then decreased. The effective penetration depth was greatest when the cone angle of the nail head was between 20° and 30°. As the nail diameter increases, the effective penetration depth decreases significantly, eventually stabilizing at a constant value. When the target sheet thickness is less than 20 mm, deformation on the reverse side of the sheet partially releases stress, reducing penetration resistance and resulting in greater penetration depth. However, when the target sheet thickness exceeds 20 mm, the penetration depth stabilizes at a constant value.

The mechanism of high-speed joining has not yet been fully elucidated. However, numerical simulations have shown that, with appropriate material models, constitutive laws, and damage laws, they can accurately predict both the sheet’s response to fastener insertion and the joint’s in-service strength. However, the challenge of numerical simulation lies in determining the correct settings for remeshing. If the frequency is too low, the mesh will degrade and the simulation will fail. If the frequency is too high, non-physical effects may occur, such as thickening of the sheet [92]. Since high-speed joining is a physical stamping process that uses kinetic energy to achieve penetration in a single step, it is limited by application scenarios. It is suitable for situations where extremely high joining speed is required and the lower plate is sufficiently strong. If the lower plate is a thin-walled cavity structure with insufficient stiffness, the impact energy will be absorbed, preventing the interlocking formation. In addition, in order to form an interlocking structure that encloses the screw, the lower plate needs to undergo severe plastic flow, which can lead to noticeable protrusions or deformations on the back side of the joint. This is extremely unfriendly for structures that require surface smoothness or have internal space interference.

### 3.4. Flow Drill Screw

The flow drill screw (FDS), also known as hot-melt self-tapping technology, is a sheet metal joining technique widely used in the high-end automotive industry [93]. The core of the FDS relies on applying high axial pressure to drive a rotating screw into the metal. The frictional heat generated by the screw’s rotation softens the sheet material locally, enabling the screw to form a threaded hole in situ. The FDS overcomes the negative impact of welding heat on joint performance [94]. By means of its outstanding single-sided joining capability, it has become the primary joining technique for sealing or semi-sealing Al extrusions and thin-walled structures.

Scholz et al. [95] investigated the influence of corrosion environments on the fatigue strength and failure behavior of FDS joints. Due to differences in the electrochemical properties of dissimilar materials, the researchers incorporated an adhesive layer at the interface, resulting in a significant improvement in joint strength and fatigue resistance. Zhou et al. [96] analyzed the grain morphology at three locations on the AA6061-T6 base sheet of the FDS joint. The microstructure at location A consists primarily of coarse grains. At location B, grains remain coarse but exhibit slight elongation. Numerous fine grains formed along the grain boundaries of elongated grains, and the microstructure at location C comprises a large number of fine equiaxed grains. The grain refinement is associated with severe plastic deformation and grain fragmentation caused by dynamic recrystallization at elevated temperatures [97].

Furthermore, FDS relies on friction-induced thermal softening, which requires time for heat accumulation. Previous studies on thermal-assisted methods have indicated that raising the pre-process temperature shortens penetration time but may extend the overall cycle. Thereby, Skovron et al. [98] explore an electrically augmented (EA) approach to reduce cycle time. The EA-FDS process is experimentally evaluated based on installation torque, process time, and breakloose torque. Results demonstrate that EA-FDS not only shortens the cycle but also enables joining of thicker cross-sections. However, electric assistance also increases the complexity of the manufacturing process. The tensile-shear strengths of joints under optimized parameters manufactured by various mechanical joining processes is shown in Table 3.

## 4. Composite Joining Technology

### 4.1. Resistance Element Welding

Resistance element welding (REW) is a composite joining technique that integrates mechanical fastening (riveting) with thermal bonding (welding) [99]. Meschut et al. [100] proposed the REW process, which achieves high-strength joining between dissimilar Al alloys and UHSS through a combination of riveting and resistance spot welding. The REW process comprises two stages: pre-setting rivets and resistance spot welding. The welding current concentrates in the element during the FEW process, generating intense resistive heat that melts the surrounding material and achieves a metallurgical joining at the dissimilar material interface [101,102]. By combining the benefits of shape fit interlocking and metallic bonding, REW leverages the existing infrastructure of conventional FSW, making it highly suitable for high-volume manufacturing.

Niu et al. [103] investigated the microstructure and mechanical performance of REW joints in AA6061-T6 Al alloy/HS1300T press hardened steel. The fusion zone (FZ) between the rivet and PHS exhibited a lath martensite structure, transformed from fast cooling of austenite. Based on microstructure and microhardness distribution, the Al side was categorized into four distinct zones: re-solidified zone (RZ), softening zone (SZ), transition zone (TZ), and base metal (BM) [104]. Cai et al. [105] employed Q235 countersunk rivets to produce REW joints between AA5052 Al alloy and DP780 dual-phase steel. The IMCs comprising Fe_2_Al_5_ and FeAl_3_ phases formed at the Al/steel interface, while the IMCs of the FeAl_3_ phase developed at the rivet/Al interface [106,107]. Three fracture modes were observed in the countersunk rivet REW joint under tensile-shear loading: interfacial failure (IF) mode, button pull-out failure (BPF) mode, and HAZ failure mode. The peak load of the joint increases with the diameter of the nugget at lower welding currents [108]. However, the peak load decreases at higher currents due to the reduction in the average hardness of the Al alloy HAZ and the bearing area of the sheet Al. The peak load of the joint can reach 5712 N, with the failure mode exhibiting the BPF mode.

To further enlarge the nugget size and improve the mechanical performance, Sun et al. [109] applied magnetic-assisted resistance element welding (MA-REW) to join AA6061 Al alloy and DP600 steel. Without raising the heat input, an annular permanent magnet induced an electromagnetic stirring effect within the nugget, thereby enlarging the nugget diameter [110]. For welding currents between 9 and 11 kA, the nugget diameter increased with the current. The external magnetic field compressed the nugget vertically while promoting lateral growth. The maximum nugget diameter of the MA-REW joints reached 3.07 mm at 11 kA. The MA-REW joints failed in the BPF mode and achieved a maximum tensile-shear load of 6674 N, a 10.2% increase compared with the REW joints that exhibited the IF mode.

Yang et al. [111] employed Q235 steel rivets to join AA6061 Al alloy with DP780 steel via the REW process and used multiple characterization techniques, including SEM and EBSD, to elucidate the morphology and formation mechanism of the IMC layer. Their findings showed that the tongue-shaped Fe_2_Al_5_ layer adjacent to the steel side grew perpendicular to the Al/steel interface, while the needle-shaped FeAl_3_ layer formed on the Al side [112,113]. The Fe_2_Al_5_ layer could be further subdivided: grains farther from the Fe layer were larger than those adjacent to it. Furthermore, dislocations were observed at the grain boundaries of Fe_2_Al_5_ grains, whereas nanotwins and stacking faults were identified within the FeAl_3_ grains, indicating that the planar defects originated primarily from rapid cooling. Compared with SPR, REW resolves the conflict between the high rigidity of steel sheets during joining and the reliance on deformation for interlocking. By achieving indirect Al/steel joining through RSW between the steel rivet and the lower sheet, REW significantly enhances joint mechanical performance. Nevertheless, the process requires an additional pre-punching step before welding, which increases equipment costs and complicates the production sequence.

IMC characterization across studies reveals both consistent findings and unresolved questions: Fe_2_Al_5_ and an Al-rich phase (FeAl_3_ or Fe_4_Al_13_) are consistently reported at the Al/steel interface. However, the exclusive formation of the Al-rich phase at the rivet/Al interface and the detailed defect structures (dislocations, nanotwins, and stacking faults) described by Yang et al. [111] have not yet been linked to crack initiation or transitions in fracture mode. The non-monotonic relationship between welding current and joint strength is widely reported, with excessive current degrading the HAZ and reducing the load-bearing area. However, systematic decoupling of the effects of thermal history, IMC growth kinetics, and HAZ softening across different alloy systems is missing. Sun et al.’s MA-REW approach alleviates this trade-off by promoting nugget growth without additional heat input, but the reported 10.2% improvement is benchmarked against REW joints that failed in the inferior IF mode, not against optimized BPF-mode REW joints. Consequently, the net benefit of magnetic stirring over a properly tuned REW process remains ambiguous. Moreover, the vertical compression of the nugget induced by the magnetic field raises questions about the through-thickness mechanical integrity and performance under multi-axial loading, aspects that have not yet been investigated.

The apparent advantage of REW over SPR—circumventing the deformation-based interlocking limitation for HSS—is partially offset by the need for a pre-punching step, which challenges the claim of seamless compatibility with high-volume manufacturing. Finally, the absence of data on fatigue life, corrosion resistance, and process robustness under production-scale variability limits the industrial transfer of these laboratory findings. Bridging these gaps will require standardized material systems and joint configurations, as well as in situ monitoring coupled with thermo-mechanical–metallurgical modeling. It will also require a holistic evaluation that integrates mechanical performance with durability and manufacturability metrics.

### 4.2. Friction Element Welding

Friction element welding (FEW) is a joining technique that utilizes friction-generated heat to soften and plastically deform the materials under stamping conditions [114]. The process combines the joining characteristics of friction spot welding (FSW) and mechanical riveting, and is employed to join dissimilar Al/steel combinations. It consists of four sequential steps: penetration, cleaning, welding, and compression. Oliveira et al. [115,116] analyzed the microstructure of FEW joints between AA6005-T5 Al alloy and Usibor1500 steel. Fine martensitic structures were observed on both sides of the joint interface, with grain refinement attributed to dynamic recrystallisation occurring in this region.

Varma et al. [117] developed a thermo-mechanically coupled finite element model of the FEW process to gain insight into the underlying physical mechanisms and to predict temperature fields and material flow during welding. The study revealed that the microhardness at the center of the nugget increased by over 50% relative to the BM, then gradually decreased across the HAZ until it reached the BM hardness level [118]. This hardness increase was closely correlated with microstructural evolution. The nugget region exhibited a significant amount of martensitic grain refinement, attributed to the internal temperature exceeding the Ac3 transformation temperature [119,120]. Additionally, high stress and severe plastic deformation at low temperatures also contributed to grain refinement.

In modern manufacturing, automated welding is increasingly replacing manual welding operations, primarily due to safety concerns associated with high temperatures, spatter, and radiation exposure. Despite its advantages, automated welding processes are susceptible to the formation of joint defects, such as porosity and cracks, which can compromise weld integrity. As automated systems lack the capability to detect such imperfections in real time, these defects may adversely affect the stability and reliability of the welding process [121,122]. Consequently, the evaluation of joint quality has become critically important in ensuring the overall performance and safety of welded structures. Antal et al. [123] indicated that pre-existing gaps between sheets critically impaired the performance of FEW joints. These gaps resulted in incomplete metallurgical bonding, excessive head protrusion, and a reduced weld area. Process optimization can reduce but cannot eliminate the peripheral edge gaps after welding, and these edge gaps can reduce load-bearing capacity and process reliability.

As can be seen from the preceding text, the FEW process is similar to the REW process. However, the two joining processes differ in several respects. Firstly, REW can be completed directly using conventional RSW equipment, whereas FEW requires the development of additional equipment. The welding cycle of REW is approximately 0.2 s. In combination with multi-degree-of-freedom robots, it can achieve high-speed assembly line operations, with process robustness superior to FEW. Secondly, REW utilizes joule heat to achieve co-melting between the auxiliary element and the lower sheet, whilst FEW uses frictional heat to bring the auxiliary element and the sheet to a thermoplastic state, achieving metallurgical bonding through atomic diffusion under axial pressure. Due to the fact that defects such as porosity and shrinkage cavities are significantly fewer in FEW joints compared to REW, the former has obvious advantages in mechanical strength and fatigue performance, and its failure mode usually manifests as ductile fracture. On the other hand, REW can only meet service requirements in lap joints of ordinary low-carbon steel thin sheets, and its failure mode usually manifests as a brittle–ductile mixed fracture. Finally, since REW involves a melting process, the high heat input leads to the formation of a thick hard–brittle IMC layer at the Al/steel interface; whereas FEW is a solid-state joining process with low and controllable heat input, which effectively suppresses the excessive growth of hard–brittle IMCs.

### 4.3. Resistance Rivet Welding

Resistance rivet welding (RRW) combines the characteristics of traditional SPR and RSW, enabling single-step joining without the need for pre-drilled holes. The process is divided into four stages: positioning, thermal piercing, welding, and retreating. Resistive heating is employed to locally bring the workpieces into a molten or plastic state, and the joint is formed upon cooling and solidification. Niu et al. [124] first proposed the RRW process, using semi-tubular rivets to join 1 mm thick AA6061-T6 Al alloy to 1.55 mm thick HS1300T PHS, and performed a detailed study of material plastic flow, elemental diffusion, and nugget growth characteristics. As the heat input increases, the nugget enlarges and the fracture mode transitions from IF to BPF. The same group later extended RRW to join a three-sheet stack consisting of 1 mm Al alloy, 1.2 mm DP780 HSS, and 1.55 mm PHS8. During welding, an Al/steel mixed nugget first forms at the Al/DP780 interface, and a steel/steel nugget develops at the DP780/PHS interface; as resistance heat accumulates, the intermediate DP780 sheet fully melts, yielding a single Al/steel/steel mixed nugget in the three-sheet joint. The maximum peak loads of the Al/steel and steel/steel interfaces reached 6213 N and 23,019 N, respectively.

**Figure 11:**
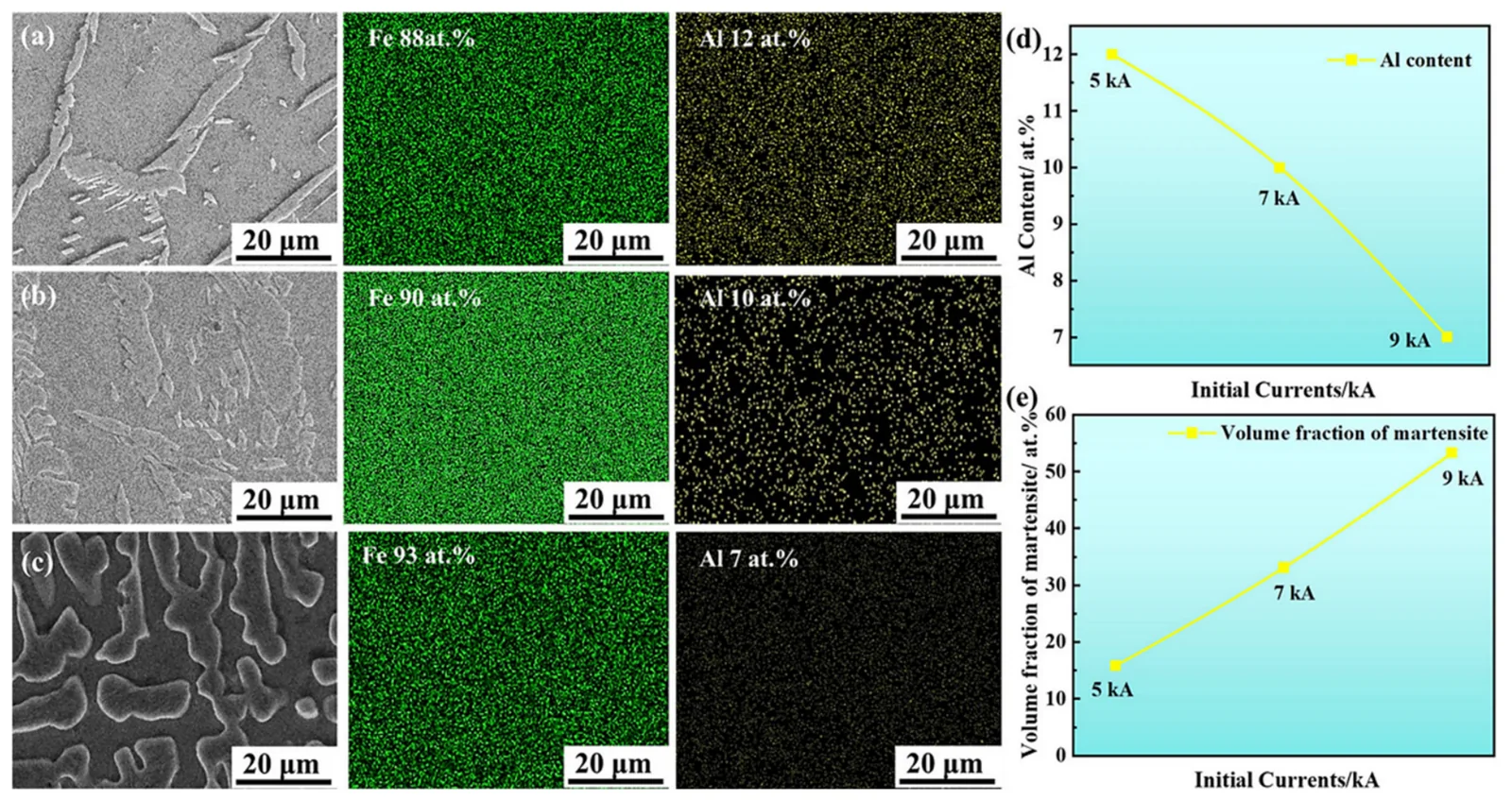
The EDS results of the mixed nugget without Mg interlayer under different heat inputs: (**a**–**c**) corresponding to the initial currents of 5 kA, 7 kA and 9 kA, respectively, (**d**) the variation in Al content, and (**e**) the volume fraction of martensite [125].

Kong et al. [125] applied the RRW process to join 2 mm thick Al alloy and 1.55 mm PHS with a semi-tubular rivet, and thoroughly elucidated the effects of a Mg interlayer on microstructure evolution, mechanical performance, and fracture behavior. At the Al/steel diffusion interface, the Mg interlayer led to a thinner IMC layer than that in joints without the interlayer because the Mg layer absorbed additional resistive heat [126]. The nugget microstructure in the absence of Mg consisted of a mixture of ferrite and martensite, whereas the Mg-bearing joints exhibited a fully martensitic structure. The mixed nugget continued to grow with increasing heat input; however, the volume of Al alloy captured within the semi-tubular rivet remained essentially constant. Consequently, the Al content in the nugget decreased from 12 at.% to 7 at.% as the initial welding current rose from 5 kA to 9 kA (Figure 11a–d). Correspondingly, the martensite volume fractions at initial currents of 5 kA, 7 kA, and 9 kA were 16%, 33%, and 53%, respectively (Figure 11e).

Due to increasingly stringent industry demands, increasingly thick cast Al components (exceeding 3 mm) are being used in Al/steel dissimilar materials joining. In order to address the aforementioned problems, Chen et al. [127] achieved high-quality flat surface joining between 3 mm thick cast Al and UHSS by adopting a presetting counter bore before the conventional RRW process. Wang et al. [128] developed the pre-piercing resistance rivet welding (PP-RRW) process to address the challenges of joining thick lightweight alloys to PHS. In this variant, resistive heat generated by a piercing electrode melts and penetrates the upper cast Al sheet. The piercing electrode is then replaced by a welding electrode to complete the conventional RRW sequence. A comparative summary of tensile-shear strengths for various composite joining processes is provided in Table 4.

While these studies collectively demonstrate the versatility and potential of RRW-based processes, a predominantly descriptive narrative leaves several interrelations, inconsistencies, and unresolved bottlenecks unaddressed. The fundamental tension lies in the claim that the process offers single-step simplicity [129,130]. For thicker or more challenging stacks, adaptations such as pre-machining a counterbore [127] or adding a separate pre-piercing stage [128] introduce extra operations that erode its stated advantage over pre-holed processes such as REW. It remains unclear whether these modified RRW variants can genuinely compete on cycle time and equipment cost.

## 5. Critical Synthesis and Future Directions

### 5.1. Joining Technologies Comparison

The reviewed processes should be evaluated according to the requirements of the intended application rather than ranked by the highest strength reported in an individual study. Mechanical joining processes, including clinching, SPR, high-speed joining and FDS joining, are attractive for sheet assembly because they avoid extensive melting and can be integrated with automated production. Their performance, however, depends on joint geometry, sheet formability, fastener or tool design, and protection against corrosion; fatigue and aging performance also require consideration. Thermal joining can provide metallurgical continuity and flexible seam formation, but the process window must balance wetting and load transfer against excessive Fe–Al intermetallic growth, porosity, and cracking. Solid-state and composite processes can reduce or redistribute thermal exposure and interfacial reaction, although specialized equipment, process control, or additional joining elements may limit their implementation. The maturity of a process should therefore be judged specifically for Al/steel joints, considering production-scale evidence, cycle time, equipment requirements, process robustness, and durability data. Reported strength values in the literature should be compared cautiously because material grades, sheet thicknesses, joint configurations, and test methods differ. These trade-offs indicate that process selection is application-dependent and should account for mechanical loading, environmental exposure, manufacturing rate, and quality-control requirements. Table 5 summarizes the principal joining approaches by their joining mechanisms, advantages, limitations, and application considerations. It complements the reported strength data by emphasizing process-level trade-offs; the strength values themselves should not be ranked directly because the cited studies use different materials, joint geometries, and test conditions.

### 5.2. Long-Term Durability and Service Reliability

Static strength alone is insufficient to assess the suitability of Al/steel joints for structural applications. Their service performance can be affected by cyclic loading, corrosive exposure, thermal cycling, and coupled thermo-electro-mechanical conditions. For example, salt-spray aging has been reported to reduce the shear and pull-out loads of EAC joints, with pull-out performance showing greater sensitivity to aging because corrosion of the sheet Al and damage to the mechanical interlock contribute to degradation [71]. Corrosion can also interact with fatigue damage in flow-drill-screw joints, while long-term coupled loading may promote interfacial diffusion and IMC growth in friction-welded joints [57,96]. These findings show that a thin initial reaction layer or high initial static strength does not by itself establish long-term reliability. Future comparisons should therefore include fatigue life, strength retention after environmental aging, failure location and mode, and the effects of relevant thermal and mechanical cycles. Because published studies use different material combinations, joint geometries, and exposure conditions, direct comparison remains difficult. Standardized durability protocols and application-relevant combined loading tests are needed to support process selection for service environments.

### 5.3. Control and Evaluation of IMCs

The effect of IMCs on Al/steel joint performance cannot be assessed from layer thickness alone. Phase composition, morphology, continuity, and spatial distribution collectively influence interfacial bonding and fracture behavior (Table 6). A thin, continuous reaction layer may provide metallurgical bonding, whereas a thick and continuous brittle layer can facilitate crack propagation along the interface. Changes in phase constitution induced by interlayers or filler elements may alter this response, but a reduction in Fe–Al IMCs does not necessarily guarantee improved joint strength if the replacement phases remain brittle or if the bonded area is reduced. Morphological features, such as tongue-shaped or needle-like phases, local discontinuities, and fragmented particles, should therefore be considered together with the location of crack initiation and the final fracture path. IMC-related brittle fracture should also be distinguished from process defects, such as porosity, hot cracking, or incomplete bonding, although these features may interact. Accordingly, the reviewed studies indicate that joint strength and failure mode depend on the combined effects of IMC characteristics, defect population, joint geometry, and loading condition.

### 5.4. Future Research Directions and Remaining Technical Challenges

Despite substantial progress in Al/steel joining, several technical challenges remain. First, future studies should establish clearer quantitative relationships among process conditions, thermal and material-flow histories, interfacial phase formation, defect evolution, and fracture behavior. This requires combining detailed interface characterization with validated thermo-mechanical–metallurgical models, rather than evaluating IMC thickness or static strength in isolation. Second, durability needs to be assessed more consistently. Fatigue, corrosion, thermal cycling, and combined service loads should be studied using representative material systems, joint geometries, and exposure conditions. Reporting fatigue life, strength retention after aging, failure location, and fracture mode would make results more comparable and help determine whether improvements in initial joint strength translate into longer service life. Third, process robustness and scale-up require further evaluation. Studies should account for production-relevant variations such as sheet gaps, surface condition, material and thickness variation, tool or electrode wear, and process repeatability. In situ monitoring and validated process models could help detect defects and define robust process windows, while cycle time, equipment requirements, and cost should be considered alongside joint performance. Finally, future comparisons would benefit from standardized test protocols and reporting practices. Because published results often involve different material grades, thicknesses, configurations, and test methods, direct ranking of processes remains difficult. A common evaluation framework covering static strength, fatigue and environmental durability, defect tolerance, process robustness, and manufacturing requirements would support more reliable technology selection for specific applications.

## 6. Conclusions

Reliable joining between Al and steel constitutes a major technological bottleneck in meeting the escalating demand for lightweight structural solutions. The underlying scientific challenges stem from residual stresses induced by mismatches in thermophysical properties, as well as the uncontrolled growth of Fe-Al IMCs at the interface. Drawing on a systematic review of thermal, mechanical, and composite joining technologies for Al/steel dissimilar systems, this study synthesizes the key conclusions and identifies future research directions.

(1) Al/steel thermal joining technologies (such as laser welding) have core advantages in terms of high energy density and strong process flexibility. Nevertheless, the laser reflectivity of Al alloys typically exceeds 80%, and the low energy utilization imposes higher requirements for further development of the process window. During solidification of the weld pool, the release of hydrogen and nitrogen can lead to porosity in the weld, and residual stresses generated by thermal deformation can also cause solidification cracks.
(2) The welding heat input directly determines the thickness of the IMC layer. If the heat input is too low, poor surface wettability may result in incomplete fusion of the joint; if the heat input is too high, an excessively thick IMC layer may cause brittle fracture of the joint. Optimizing process parameters, adding filler materials and intermediate layers, and setting external energy fields can effectively control the thickness of the IMC layer at the interface of RSW welds and improve the mechanical properties of Al/steel welded joints.
(3) Al/steel mechanical joining technologies facilitate the dissimilar materials joining via plastic deformation or mechanical interlocking, thereby precluding the formation of Fe-Al IMCs. Clinching has lower production costs because it does not require additional fasteners such as rivets. SPR does not require pre-drilling holes and are distinguished by the high productivity. However, challenges such as insufficient interlocking quantity, sheet steel fracture, and fasteners upsetting may be encountered during the piercing of UHSS sheets. High-speed joining and FDS, as widely implemented single-sided fastening technologies, are particularly advantageous for single-sided accessible joining scenarios in closed cavity structures.
(4) The interlocking of the clinching joints increases with the increasing pre-forming depth, whereas the neck thickness is mainly influenced by the die depth. The forming quality of the SPR joints mainly depends on structural parameters such as rivet size, electrode shape, and die depth, as well as process parameters such as electrode pressure, welding current, and welding time. Systematically optimizing the above parameters through multi-objective optimization methods and establishing finite element forming models can further improve the mechanical performance of the joints.
(5) Al/steel composite joining technologies such as REW transform the Al/steel dissimilar joining into steel/steel homogeneous joining by introducing additional components. By combining the advantages of form fit and metallurgical bonding, these methods overcome the limitations of conventional welding techniques, including the difficulty of controlling IMCs and the challenges of penetrating HSS. Although FEW offers considerable joining performance, the development of entirely new equipment entails high costs. To further simplify the process and accelerate production cycles, various derivative processes based on REW and RRW provide new approaches for Al/steel dissimilar material joining.

## Figures and Tables

**Table 1:** Comparison of thermophysical properties between Al and steel [21].

Thermophysical Properties	Al	Fe
Specific Heat (J·kg^−1^·K^−1^)	1080	795
Melting Point (°C)	660	1536
Boiling Point (°C)	2520	2860
Density (kg/m^−3^)	2385	7015
Resistivity (10^−8^ Ω·m)	2.82	9.71
Yield Strength (MPa)	7~11	150~200
Tensile Strength (MPa)	80~100	250~330
Elastic Modulus (GPa)	70	210
Linear Expansion Coefficient (10^−6^/°C)	24	12
Thermal Conductivity (W/(m·K))	237	46

**Table 2:** Mechanical properties of Al/steel joints produced by different thermal joining technologies.

Material Type and Sheet Thickness	Process	Reported Tensile-Shear Result	Ref.
6061 (2 mm)/316L (2 mm)	LAHW	224.8 MPa	[25]
6061 (1.5 mm)/DP980 (1 mm)	LSFW	4.1 kN	[29]
5052 (2 mm)/Q235B (2 mm)	LAHW	195.6 MPa	[30]
6061 (1 mm)/DP590 (1 mm)	MA-LBW	0.9 kN	[31]
6061 (2 mm)/DP590 (2 mm)	LWB	210.7 MPa	[33]
5052 (2 mm)/GS (2 mm)	BF	1.5 kN	[34]
6061 (2 mm)/DP590 (2 mm)	LWB	134.1 MPa	[35]
6061 (2 mm)/DP590 (2 mm)	LWB	225.6 MPa	[36]
X626 (0.8 mm)/LCS (1 mm)	RSW	3.1 kN	[38]
6022 (1.2 mm)/HSLA (2 mm)	RSW	7.0 kN	[39]
6061 (1.5 mm)/DP590 (1.5 mm)	LAE-RSW	5.8 kN	[40]
5754 (2 mm)/Q235 (1.8 mm)	CMT	65.0 MPa	[44]
6022 (1.2 mm)/HSLA (2 mm)	RSW	98.0 MPa	[45]
6061 (1.5 mm)/DP800 (1.5 mm)	USW	3.4 kN	[59]
2024 (2 mm)/HSLA (1.2 mm)	USW	6.8 kN	[68]

**Table 3:** Mechanical properties of Al/steel joints produced by different mechanical joining technologies.

Material Type and Sheet Thickness	Process	Reported Tensile-Shear Result	Ref.
5052 (2 mm)/DZ52D (1.5 mm)	EAC	3.6 kN	[70]
7075 (2 mm)/22MnB5 (1.5 mm)	S-C	4.1 kN	[73]
5182 (2 mm)/HC340LA (2 mm)	E-SPR	6.8 kN	[76]
6061 (2 mm)/DP980 (1.2 mm)	TA-SPR	3.8 kN	[78]
6063 (2 mm)/DP590 (0.8 mm)	GA-SPR	6.5 kN	[79]
6063 (3 mm)/DP780 (1.2 mm)	SPR	7.9 kN	[81]
6061 (1.5 mm)/DP980 (1.5 mm)	R-SPR	3.6 kN	[82]
6061 (2.7 mm)/DP590 (1.8 mm)	FDS	7.9 kN	[96]

**Table 4:** Mechanical properties of Al/steel joints produced by different composite joining technologies.

Material Type and Sheet Thickness	Process	Reported Tensile-Shear Result	Ref.
6016 (1.2 mm)/22MnB5 (1.5 mm)	REW	3.3 kN	[99]
6061 (1 mm)/PHS (1.5 mm)	REW	5.1 kN	[103]
5052 (2 mm)/DP780 (2 mm)	REW	5.7 kN	[105]
6061 (2 mm)/DP600 (1.5 mm)	MA-REW	6.7 kN	[109]
6061 (1.5 mm)/DP980 (1.4 mm)	FEW	10.1 kN	[119]
6061 (1 mm)/PHS (1.5 mm)	RRW	5.1 kN	[124]
6061 (2 mm)/PHS (1.5 mm)	RRW	8.5 kN	[125]
JDA1B (3 mm)/PHS (1.5 mm)	RRW	10.7 kN	[127]
C611 (3 mm)/PHS (1.5 mm)	PP-RRW	10.2 kN	[128]

**Table 5:** Comparative summary of Al/steel joining technologies.

Joining Approach and Examples	Main Joining Principle	Key Advantages	Main Limitations and Trade-Offs	Application Considerations
Thermal joining: fusion welding, laser welding–brazing, RSW, FSW and related variants	Forming metallurgical bonds through localized melting, wetting, diffusion, pressure, solidification, frictional heating and plastic flow	Providing metallurgical bonding; achieve flexible seam or spot connections, reduce excessive melting, and limit the formation of thick IMCs	Thermal joining processes require careful control of heat input to balance bonding against IMC growth, porosity, cracking, and HAZ changes	Suitable where metallurgical bonding is required; select the specific process according to joint geometry, access, material stack, thermal sensitivity, and production requirements
Mechanical joining: clinching, SPR, FDS, high-speed joining	Plastic deformation and mechanical interlocking, with no direct metallurgical Fe–Al bond	Avoids fusion-related IMCs and heat damage; several methods support rapid or automated sheet assembly	Performance depends on sheet formability, interlock geometry, fastener/tool design, and corrosion protection; fatigue and aging behavior require consideration	Useful when low heat input or one-sided access is needed; select according to load direction, sheet stack, and access constraints
Composite joining: REW, FEW, RRW and related variants	Combines mechanical interlocking or an auxiliary element with metallurgical bonding	Can extend joining options for high-strength steel stacks and combine load-transfer mechanisms	May require extra components, pre-punching, specialized equipment, or tighter process control; added operations can affect cost and cycle time	Consider when a single joining mechanism cannot meet stack or load requirements; assess production robustness and equipment needs

**Table 6:** Relationship between IMC characteristics and joint performance.

IMC Characteristics	Potential Influence on Joint Behavior	Factors to Consider in the Assessment
Phase composition	Different Fe–Al phases or modified reaction products may affect interfacial brittleness and the location of fracture.	Phase identification, interfacial bonding integrity, and the actual fracture path
Morphology and continuity	A continuous layer may provide a path for interfacial crack propagation, whereas local discontinuities or particulate phases may alter crack propagation.	Areal coverage, thickness uniformity, and the presence of associated cracks or pores
Layer thickness	An excessively thick, continuous reaction layer may increase the tendency for brittle fracture. Conversely, an excessively thin or locally absent layer may be associated with inadequate bonding.	Heat input, effective bonded area, joint geometry, and loading mode
Spatial distribution and local defects	Local IMC accumulation or interfacial heterogeneity may interact with stress concentration, crack initiation, and crack propagation.	Porosity, hot cracking, unbonded regions, heat-affected-zone softening, and failure location

## Data Availability

No new data were created or analyzed in this study. Data sharing is not applicable to this article.
